# Revisiting Warfarin Dosing Using Machine Learning Techniques

**DOI:** 10.1155/2015/560108

**Published:** 2015-06-04

**Authors:** Ashkan Sharabiani, Adam Bress, Elnaz Douzali, Houshang Darabi

**Affiliations:** ^1^Department of Mechanical and Industrial Engineering, University of Illinois at Chicago, Room 4209, SEL West Building, 950 South Halsted Street, Chicago, IL 60607, USA; ^2^Department of Pharmacotherapy, University of Utah, 30 South 2000 East, Room 4929, Salt Lake City, UT 84112, USA; ^3^Department of Mechanical and Industrial Engineering, University of Illinois at Chicago, Room 2055, ERF Building, 842 W Taylor Street, Chicago, IL 60607, USA

## Abstract

Determining the appropriate dosage of warfarin is an important yet challenging task. Several prediction models have been proposed to estimate a therapeutic dose for patients. The models are either clinical models which contain clinical and demographic variables or pharmacogenetic models which additionally contain the genetic variables. In this paper, a new methodology for warfarin dosing is proposed. The patients are initially classified into two classes. The first class contains patients who require doses of >30 mg/wk and the second class contains patients who require doses of ≤30 mg/wk. This phase is performed using relevance vector machines. In the second phase, the optimal dose for each patient is predicted by two clinical regression models that are customized for each class of patients. The prediction accuracy of the model was 11.6 in terms of root mean squared error (RMSE) and 8.4 in terms of mean absolute error (MAE). This was 15% and 5% lower than IWPC and Gage models (which are the most widely used models in practice), respectively, in terms of RMSE. In addition, the proposed model was compared with fixed-dose approach of 35 mg/wk, and the model proposed by Sharabiani et al. and its outperformance were proved in terms of both MAE and RMSE.

## 1. Introduction

A great deal of effort has been dedicated to determine the optimal initial dose for warfarin. The challenge in estimating the right dose of warfarin for each patient arises from the fact that there is wide interpatient variability in dosing [1]. Over the past decade or so, a number of research groups have focused on developing models to predict the warfarin maintenance dose. Accurate warfarin dosing is critically important because of the drug's narrow therapeutic index, whereas there is an increased risk for thromboembolism or hemorrhage with sub- or supratherapeutic anticoagulation, respectively. In particular, the risk for bleeding increases when the international normalized ratio (INR) surpasses 3 [2], while the risk for thrombosis increases when the INR falls below 2 [3]. As a result, warfarin is the leading cause of drug-related hospitalizations among older adults in the United States of America [1]. The risks for bleeding or thrombosis with warfarin are greatest during the initial months of therapy [1]. Therefore, selecting an appropriate dose at the initiation of therapy is important to achieve optimal anticoagulation to reduce adverse effects. An additional challenge with warfarin dosing is the significant variability amongst patients in the dose required for therapeutic anticoagulation. Clinical factors, including age, body size, and use of medications that affect warfarin metabolism, contribute to warfarin dose requirements [4, 5]. In addition, genes involved in warfarin metabolism and determining warfarin sensitivity, namely, the cytochrome P450 2C9 (CYP2C9) and vitamin K epoxide reductase complex 1 (VKORC1) genes, significantly impact warfarin dose requirements. A recent clinical trial in a predominantly European population showed that the use of a pharmacogenetic model, containing genotype plus clinical factors, was superior to conventional warfarin dosing [6]. However, another trial in a more ethnically diverse population showed no benefit with a pharmacogenetic model versus a clinical model, containing just clinical factors [7]. Previous studies have shown better warfarin dose prediction with a clinical dosing algorithm versus convention dosing (e.g., fixed dose of 5 mg/day).

The proposed prediction models range from traditional methods such as linear regression modelling to more advance models which belong to the class of machine learning techniques.

In 2008 Gage et al. proposed 2 linear regression models involving pharmacogenetic and clinical factors to predict the therapeutic dose of warfarin. They applied BSA (body surface area) instead of height and weight, used the actual age values and not age categories, and also involved “Smokes,” “Target INR,” and “DVT/PE” (deep vein thrombosis or pulmonary embolism) in their models [4]. They trained their models in 1015 patients and tested them in 292 patients. In 2009, the IWPC (International Warfarin Pharmacogenetics Consortium) research team gathered patients' data of different ethnicities, 21 various research groups, 9 countries, and crossing 4 continents on warfarin-treated patients, totaling 5052 number of patients. After investigating several prediction models such as ordinary linear and polynomial regression, artificial neural networks (ANN), support vector regression with polynomial (including linear) and Gaussian kernels, regression trees, model trees, least angle regression, and Lasso and multivariate adaptive regression, they proposed 2 linear regression models (a clinical and one pharmacogenetic model). The variables involved in the proposed models differ from the Gage's models from different aspects. Instead of BSA, the actual values for height and weight were used. Instead of the real values for age, the age decade was used. “Smokes,” “Target INR,” and “DVT/PE” were not applied in the models. They claimed that the clinical model is well suited for patients requiring doses between 21 and 49 mg/week [5]. The abovementioned models are the most recommended models for determination of the initial warfarin dose according to the “Clinical Pharmacogenetics Implementation Consortium Guidelines for CYP2C9 and VKORC1 Genotypes and Warfarin Dosing” [8]. In 2014, Grossi et al. proposed a new prediction model using artificial neural network. Using the data of 377 patients, they selected 23 variables by TWIST system and derived an ANN model [9]. They proved that the proposed model outperformed those of IWPC [5], Gage et al. [4], and Zambon et al. [10] in terms of mean absolute error (MAE) and model's fitness (*R* ^2^). Furthermore, several models have been proposed for specific ethnicity groups, different age groups, or geographical areas. In 2011, Cosgun et al. proposed three pharmacogenetic prediction models using machine learning approaches for African-American patients. The models were random forest regression (RFR), boosted regression tree (BRT), and support vector regression (SVR) [11]. They used *R* ^2^ as the index for predictive accuracy and claimed that their model outperformed previously proposed pharmacogenetic models, namely, Limdi et al.'s [12, 13] and Schelleman et al.'s models [14, 15]. In 2013, Sharabiani et al. proposed a new clinical model for African-American patients. The proposed model outperforms IWPC and Gage models in terms of prediction accuracy [16]. Hernandez et al. also proposed a pharmacogenetic model customized for African-American patients. They compared their model with IWPC pharmacogenetic and clinical models and proved their model's outperformance [17].

Monagle et al. investigated the impact of pharmacogenetics-based warfarin dosing in children. Despite the presence of multiple prediction models for adults, not many models are available for children. The most simple dosing procedure for children is the weight-based dose model with initial dose of 0.2 mg/kg/day [18]. In addition, several models have been proposed in the literature which are solely designed for children, such as models proposed by Nowak-Göttl et al. [19], Moreau et al. [20], Biss et al. [21], Nguyen et al. [22], and Kato et al. [23]. The proposed models also took advantage of the pharmacogenetic factors along with the clinical factors.

Despite the application of pharmacogenetic factors in the proposed models, the application of pharmacogenetic factors in prediction models is still a controversial issue. Burmester et al. compared the time to reach the therapeutic dose on two patient cohorts. They established the initial dose solely by clinical factors for the first group and added the pharmacogenetic factors for the second cohort. They claimed that involving the pharmacogenetic factors did not make any significant difference in reaching the time to the therapeutic dose. This study is known as “Marshfield Clinic Research Foundation (MCRF)” [24]. Stergiopoulos and Brown also investigated the difference between genotype guided versus clinical dosing of warfarin. They also proved that, in meta-analysis of randomized clinical trials, a pharmacogenetic dosing method did not cause a superior percentage of time that the INR fell within the therapeutic range [25]. In spite of encouraging research outcomes and US FDA warfarin label adjustments, the Centers for Medicare and Medicaid Services (CMS) have not regularly enclosed clinical CYP2C9 and VKORC1 genotyping, and therefore it demands additional evidence to require the need for genotyping. In addition to MCRF, several European research teams also made inquiries on the impact of pharmacogenetic factors on warfarin dosing, such as CoumaGen [26], CoumaGen-II [7], European Pharmacogenetics of Anticoagulant Therapy (EU-PACT) [6], and Clarification of Optimal Anticoagulation Through Genetics (COAG) [27]. Most of the abovementioned studies do not claim a general conclusion on accepting or rejecting the pharmacogenetic models. For example, the EU-PACT demonstrates that “pharmacogenetic-guided dosing is superior to a fixed-dosing regimen for achieving therapeutic international normalized ratios in Caucasian patients initiated on warfarin.” For the detailed comparison on different studies or challenges on involving the pharmacogenetic factors on warfarin dosing, see [28, 29].

Considering the prevailing uncertainty of applying the pharmacogenetics-based models and the fact that, in practice, the availability of gene information may be limited, and hence not many clinicians have access to that data; in this paper we have concentrated on developing a dose prediction methodology using only clinical factors.

In this paper, a novel methodology towards warfarin dosing for adults is proposed using the clinical variables. In this methodology, initially, the patients get classified into two classes. The first class is the patients who require doses of >30 mg/wk and the second class contains the patients who require doses of ≤30 mg/wk. In the following phase, the optimal dose for each patient will be predicted by two regression clinical models which are customized for each class of patients. The proposed methodology is proven to outperform the existing popular clinical prediction models in terms of prediction accuracy.

## 2. Materials and Methods

### 2.1. The Dataset

The dataset that we have used in this paper is the IWPC dataset which is a well-known multiethnic warfarin dataset. This dataset is one of the most widely used and publically available warfarin datasets, as evident by its citations in the literature [30]. We handled the missing values in the dataset by imputation using the *K*-nearest neighbor (KNN) method with *k* = 1 [31]. The variables whose percentage of missing values was more than 50% were not involved in the model. The variables used in the modeling were only the clinical and demographic variables which are presented in Table 1. In order to develop a robust prediction model, we followed the CRISP-DM methodology in order to build our models [32]. We randomly selected 50% of the data points to comprise the training set (derivation cohort) and the remaining 50% were assigned to the testing set (validation cohort). The data in the test set was used for the models' performance in dealing with unseen data points.

### 2.2. The Proposed Methodology

The dose prediction method that is proposed in this paper contains two phases. In the first phase, the data points in the test will be assigned to two classes. The first class contains patients who require doses of >30 mg/wk (high required dose (HRD)) and the second class contains the patients who need doses of ≤30 mg/wk (low required dose (LRD)).

The selected cut-off point (30 mg/wk) was derived from the validation process in which the data in the learning set was divided randomly into training and validation sets. Different values (15, 20, 30, 35, 40, 45, and 50 mg/wk) were selected and examined to identify the threshold that maximized the classification accuracy. The optimal threshold, 30 mg/wk, from the validation process, was applied in the modelling procedure.

**Figure 1:**
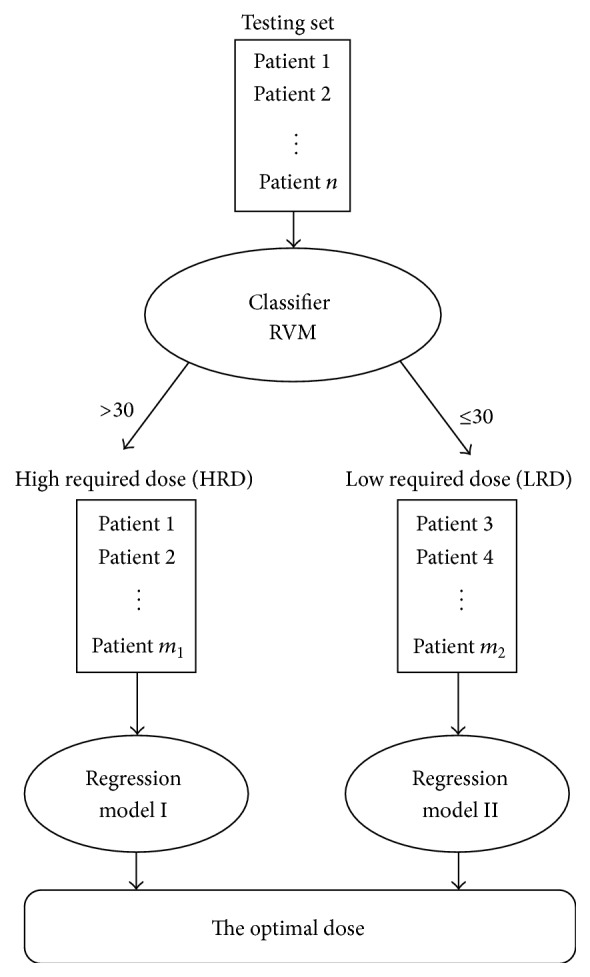
The proposed methodology.

This phase is performed using a classification technique which incorporates relevance vector machines (RVM). In the second phase, the optimal dose for each patient will be predicted by two regression clinical models which are customized for each class of patients; see Figure 1.

### 2.3. Training the Models

The classification and the regression models are created using the data points in the learning set. Each data point in the learning set got labeled as 0 (LRD patients) or 1 (HRD patients) depending on the value of the therapeutic dose. Now by considering the generated labels as the new response variable, the nature of the problem transforms to classification. A classification model (RVM) is trained using the data in the learning set. Additionally, the points in the learning set are assigned to two groups according to their label and a regression model for each group gets generated.

As it is shown in Figure 1, when the points are labeled as 1 or 0 by the classification model, they will get entered into the second phase which is the prediction phase. A comprehensive review on machine learning methods and, specifically, support vector machines and relevance vector machines are presented in the next section.

### 2.4. Machine Learning

Machine learning (ML) is known as a branch of artificial intelligence. The major goal in ML is developing models and techniques that enable the computers to learn. The methods in ML can be categorized into two broad categories: supervised and unsupervised techniques. The difference between these techniques is the presence of response variables in the dataset. Therefore, once the response variable is unknown, the nature of the problem calls for unsupervised methods such as clustering. Subsequently, when the response variable is known, supervised methods will come into practice. If the response variable is known and takes numerical values, prediction models will be used, such as regression, and when it takes categorical values, classification models will be applied [31]. Several powerful classification models have been developed in the last 6 decades, namely, decision tree [33], artificial neural network [34], support vector machines [35], logistic regression [36], and so forth.

### 2.5. Support Vector Machines

As discussed above, since we aim to classify patients to either class HRD or class LRD in the initial phase of the modeling, our problem is a classification problem. Among numerous classifiers that are proposed in machine learning literature, support vector machine (SVM) is one of the most popular classification techniques. This model was first introduced by Vapnik in 1998 [37]. SVMs use a simple linear method applied to the data but in a high-dimensional feature space which is nonlinearly associated with the input space [30].

In a typical classification problem, the dataset consists of several features *X*_1_, *X*_2_,…, *X*_*L*_ and one or several variables for labels *C*_1_, *C*_2_,…, *C*_*p*_. The goal is to develop a model to assign the objects (data points) to their classes. The classification model that was used in this paper is relevance vector machines (RVM) which is a special form of support vector machines (SVM). In a two-class classification problem (*C*_1_ and *C*_2_), the objective is to develop a classifier using the *N* data points in the training set. Therefore for each point in the training set {*x*_*n*_}_*n*=1_^*N*^ a label *z*_*n*_ ∈ {−1,1}, *n* = 1,…, *N* should be estimated. The classifier is defined as

**Figure 2:**
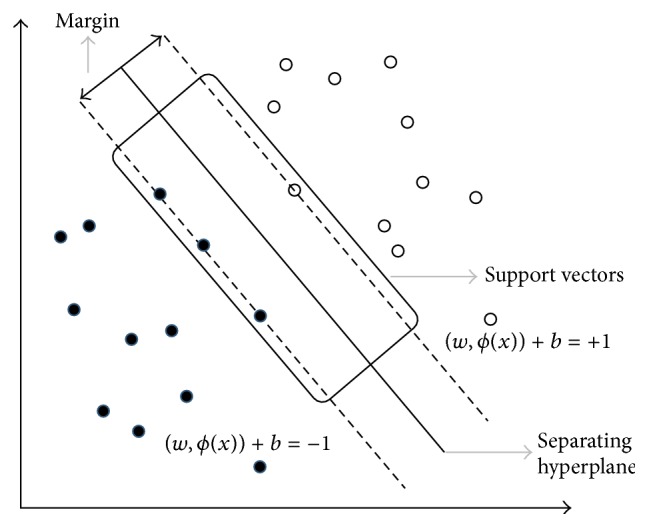
The separating hyperplane.

(1) yx;w≜wTϕx+bor  yx;w≜∑i=1Mwiϕix+b,

where *w* ∈ *R* ^*M*^ is the weight vector, *b* ∈ *R* is the constant, and *ϕ*(·) is the transformation function. The predicted labels are computed using the sgn⁡(·) function, sgn⁡(*y*(*x*)). Assuming the data is linearly separable, there exist vectors *w*(*w* ^*∗*^) and *b*(*b* ^*∗*^) which yield a* hyperplane* that completely separates the data to two disjoint areas. This* hyperplane* is called the decision boundary (*D*) and the predicted labels for the data points and the value of *y*(*x*_*n*_) have the same sign (*z*_*n*_ *y*(*x*_*n*_) > 0; ∀*x*_*n*_ ∈ *R* ^*D*^ and *z*_*n*_ ∈ {−1,1}). The minimum distance of the points in the training set to *D* is called the* margin* (see Figure 2) which is computed using min_*n*∈{1,…,*N*}_(*z*_*n*_ *y*(*x*_*n*_)/‖*w*‖); ‖·‖ is the *L* ^2^-norm. The objective in SVM is choosing the values for *W* and *b* which maximizes the* margin*. The values for *w* ^*∗*^ and *b* ^*∗*^ are yielded by solving the following optimization problem:

(2) $$\begin{array}{c} \underset{w\in R^{M},b\in R}{\max}\left\{\;\frac{1}{\left\|w\right\|}\underset{n\in \left\{1,\ldots ,N\right\}}{\min}\left[\;z_{n}\left(w^{T}\phi \left(x_{n}\right)+b\right)\;\right]\;\right\}. \end{array}$$

The *w* ^*∗*^ and *b* ^*∗*^ which resulted from (2) are also the solutions to the following minimization problem:

(3) minw∈RM,b∈R 12w2subject  to znwTϕxn+b≥1,

where *x*_*n*_ ∈ *ℝ* ^*D*^, *z*_*n*_ ∈ {−1,1}, and *n* = 1,…, *N*.

The optimization problem in (3) can also be solved by applying Lagrange multipliers (*λ*_*n*_ ∈ *R*, *n* = 1,…, *N*). The Lagrangian formation of (3) is

(4) Lw,b,λ=12w2−∑n=1NλnznwTϕxn+b−1.

The first-order conditions for optimality in (4) are ∑_*n*=1_^*N*^ *λ*_*n*_ *z*_*n*_ *ϕ*(*x*_*n*_) = *w* and ∑_*n*=1_^*N*^ *λ*_*n*_ *z*_*n*_ = 0. After applying the conditions, the dual form of (3) will result in

(5) maxλ∈RN Lλsubject  to λn≥0,n=1,…,N ∑n=1Nλnzn=0,

where *ℒ*(*λ*)≜∑_*n*=1_^*N*^ *λ*_*n*_ − (1/2)∑_*n*=1_^*N*^∑_*m*=1_^*N*^ *λ*_*n*_ *λ*_*m*_ *z*_*n*_ *z*_*m*_ *k*(*x*_*n*_, *x*_*m*_) and *k*(*x*, *x*′) = *ϕ* ^*T*^(*x*)*ϕ*(*x*′) are called the kernel function. The KKT (Karush-Kuhn-Tucker) conditions for optimality for optimization problems in (3) and (5) are *λ*_*n*_ ≥ 0, *z*_*n*_ *y*(*x*_*n*_) − 1 ≥ 0, and *λ*_*n*_(*z*_*n*_ *y*(*x*_*n*_) − 1) = 0, where *n* = 1,…, *N*. Those data points for which the corresponding *λ*_*n*_ is nonzero are called* support vectors*. These points play a crucial role in classifying new points.

If the points in the dataset are not linearly separable, by using slack variables (*ξ*_*n*_ ≥ 0) the concept of soft-margin classifiers will be defined. In this family of classifiers, by assigning a penalty to the points that lay on the wrong side of the boundary, the optimization problem in 3 will be rewritten as follows:

(6) minw∈RM,b∈R,ξ∈RN C∑n=1Nξn+12w2subject  to znyxn≥1−ξn,n=1,…,N ξn≥0,n=1,…,N.

*C* > 0 is called the* complexity parameter*. The Lagrangian method can again be applied for solving (6) which has the form *ℒ*(*w*, *b*, *λ*, *ξ*) = (1/2)‖*w*‖^2^ + *C*∑_*n*=1_^*N*^ *ξ*_*n*_ − ∑_*n*=1_^*N*^ *λ*_*n*_(*z*_*n*_ *y*(*x*_*n*_) − 1 + *ξ*_*n*_) − ∑_*n*=1_^*N*^ *μ*_*n*_ *ξ*_*n*_, where *w* = ∑_*n*=1_^*N*^ *λ*_*n*_ *z*_*n*_ *ϕ*(*x*_*n*_), 0 = ∑_*n*=1_^*N*^ *λ*_*n*_ *z*_*n*_, *λ*_*n*_ = *C* − *μ*_*n*_, *n* = 1,…, *N*, and *λ*_*n*_ ≥ 0. The dual form of this optimization problem is presented in

(7) minλ∈RN Lλsubject  to 0≤λn≤C,n=1,…,N ∑n=1Nλnzn=0.

The major drawbacks of SVM are as follows.

- The linear growth of the number of support vectors is with the number of data points in the training set.
- Providing a hard binary decision, in most applications it would be much more useful when the level of certainty is addressed when classifying new objects.
- It is necessary to estimate the *C* (complexity parameter) which requires the cross-validation.

To overcome the abovementioned shortcomings, in the next section the relevance vector machines (RVM) will be introduced.

### 2.6. Relevance Vector Machines

Relevance vector machines (RVM) belong to the family of sparse Bayesian learners. This method, which can be used for both classification and regression, was introduced by Tipping [38]. One of the most important advantages of RVMs is its ability for handling classification problem when the cost of misclassification includes different classes. In a classification problem, RVM assigns a class membership probability for a given point (*x*): *p*(*C*_*k*_∣*x*, *X*, *Z*), where *X* is the feature set and *Z* is the set of labels in the training set. Assuming that the posterior probability of a target variable in *C*_1_ is calculated by

(8) $$\begin{array}{c} p\left(\;z_{n}=1\;|\;x_{n},w\;\right)=\frac{1}{1+e^{-\left({x_{n}}^{T}\phi \left(x\right)+b\right)}},\;n=1,\ldots ,N, \end{array}$$

we will configure the likelihood function (LF). Using *σ*(·) for the logit function, the right side of (8) can be denoted as *σ*(*y*(*x*_*n*_)). Therefore, in our binary classification problem, the LF is

(9) pZ ∣ X,w∏n=1Npz ∣ xn,w=∏n=1Nσyxnzn1−σyxn1−zn.

The weight parameters (*w*) in (9) have a Gaussian distribution with a mean of zero. However the variance of each *w*_*i*_ *i* = 1,…, *M* could be different. So, the prior distribution of the weight vector will be

(10) $$\begin{array}{c} p\left(\;w\;|\;\alpha \;\right)=\overset{M}{\underset{n=1}{\prod }}N\left(\;w_{n};0,\alpha_{n}^{-1}\;\right), \end{array}$$

where *α*_*i*_, *i* = 1,…, *M* is known as hyperparameters and is the inverse of the Gaussian distribution variance. For any new point (*x*) the posterior probability can be calculated as *p*(*z*∣*x*, *X*, *Z*). This probability is computed by marginalizing the *p*(*z*, *x*, *X*, *Z*, *w*, *α*):

(11) pz ∣ x,X,Z=∬−∞∞pz ∣ x,X,Z,w,α×pw ∣ x,X,Z,αpα ∣ x,X,Zdw dα.

Solving (11) can be done by using approximation, in which for the vector of *α* we will use a constant (*α* ^*∗*^). *α* ^*∗*^ is the value which maximizes the *p*(*Z*∣*X*, *α*). Therefore, (11) will be equal to

(12) $$\begin{array}{c} \overset{\infty }{\underset{-\infty }{\int }}p\left(\;z\;|\;x,X,Z,w,\alpha^{*}\;\right)p\left(\;w\;|\;x,X,Z,\alpha^{*}\;\right)dw. \end{array}$$

Furthermore, *p*(*w*∣*x*, *X*, *Z*, *α*) = *p*(*Z*∣*x*, *X*, *w*, *α*)*p*(*w*∣*x*, *X*, *α*)/*p*(*Z*∣*x*, *X*, *α*) = *p*(*Z*∣*X*, *w*)*p*(*w*∣*α*)/*p*(*Z*∣*X*, *α*). This probability should also get approximated. The approximation process aims to detect the vector of *w* which maximizes *p*(*w*∣*x*, *X*, *Z*, *α*). The maximization problem (*w* ^*∗*^) is

(13) $$\begin{array}{c} \underset{w\in R^{M}}{\max}\left\{\;\ln\left(\;p\left(Z\;|\;X,w\right)p\left(w\;|\;\alpha \right)\;\right)-\ln p\left(Z\;|\;X,\alpha \right)\;\right\} \end{array}$$

and the marginal LF *p*(*Z*∣*X*, *α*) will be

(14) ∫−∞∞pZ ∣ X,w,αpw ∣ X,αdw=∫−∞∞pZ ∣ X,wpw ∣ αdw

which, using the Laplace approximation method, is equivalent to

(15) $$\begin{array}{c} p\left(\;Z\;|\;X,w^{*}\;\right)p\left(\;w^{*}\;|\;\alpha \;\right){\left(\;2\pi \;\right)}^{N/2}{\left(\;\det\Sigma \;\right)}^{1/2}. \end{array}$$

The Σ in (15) is the covariance matrix of the Gaussian approximation. Using the approximation method, the vector of *α* and *w* will be estimated. Surprisingly enough, the value of *α* for most weights goes to infinity which will result in minimizing *w* to zero. Therefore, this process will yield a much sparser model. The points in the training set for which the corresponding *w* is nonzero are called the relevance vectors.

## 3. Evaluation Methods

There are several methods to evaluate a classification method. In Table 2, the fundamental definitions for a confusion matrix are presented. A confusion matrix is a tabulated presentation of correctly or incorrectly classified points in the dataset. The definition of the cell values in the confusion matrix is presented below:

- true positives (TP): the number of positive examples that were predicted correctly,
- false positives (FP): the number of positive examples that were predicted incorrectly,
- true negatives (TN): the number of negative examples that were predicted correctly,
- false negatives (FN): the number of negative examples that were predicted incorrectly.

The measures that were considered to pick the best model are as follows:

(16) Accuracy=TP+TNTP+TN+FP+FN,Sensitivity=TPTP+FN,Specificity=TNTN+FP,Precision+=TPTP+FN,Precision−=TNTN+FP.

In the next section, the experimental results for applying the proposed methodology on the dataset will be presented.

## 4. Results and Discussion

Using the RVM model, the data points in the testing set were classified to HRD and LRD classes and two regression models were developed for each class separately. The models are presented below.

Model for HRD class (Model I):

(17) Predicted  Dose=Exp2.85332−0.07370×Race−0.06513×Age+0.10246×DVTPE+0.05766×Diabetes+0.03742×VR−0.08763×Lovastatin−0.12542×Amiodarone+0.13207×TargetINR+0.12403×Enzyme+0.34487×BSA.

Model for HRD class (Model II):

(18) Predicted  Dose=Exp3.44056−0.03649×Race−0.04820×Age+0.05059×DVTPE−0.03060×Aspirin−0.06150×Amiodarone−0.20356×AfungalAzoles+0.05744×Smoker+0.10923×Enzyme+0.24601×BSA.

In the cross-validation phase, the trained models were applied on the data points in the testing set. The classification results for the two models are presented in Table 3.

After classifying the points in the test set, 49% of the points were assigned to HRD class and 51% to LRD class. The proposed method's prediction accuracy got evaluated based on RMSE (root mean squared error): $\sqrt{mean[{\left(Actual\;\;Value-Predicted\;\;Value\right)}^{2}]}$ and MAE (mean absolute error): mean (|Actual  Value − Predicted  Value|). The prediction results are presented in Table 4.

As it is evident in Table 4, the proposed methodology for predicting the warfarin dose outperforms the IWPC cl model for 16% in terms of RMSE and 8% in terms of MAE. It also outperforms the Gage Cl model for 5% in terms of RMSE and 16% in terms of MAE. The proposed method was also compared with fixed-dose approach (35 mg/wk) and the prediction model proposed in [16]. The method resulted in significantly lower RMSE and MAE than both models (37%, 31% less than the fixed-dose approach and 35%, 33% less than the method in [16] in terms of RMSE and MAE, resp.). In Table 4, we have compared our methods with four other clinical methods that are either widely used or have outperformed other widely used models. We were not able to find any other clinical model in the literature that has an advantage (either in terms of popularity or in terms of prediction accuracy) over these selected methods. Therefore, our conclusion is that our proposed method outperforms all available clinical models for initial warfarin dosing in the literature.

We have not compared our model with any existing pharmacogenetic model (e.g., the models proposed in [9, 11]). As we mentioned in the Introduction section, there is no general consensus in the literature that pharmacogenetic models outperform clinical models. Even if pharmacogenetic models had generally a higher accuracy of warfarin dose prediction, such a comparison would have not been absolutely required due to the differences in the application domains of these classes of models. In practice, for some patients, it is impossible to use a pharmacogenetic model. Pharmacogenetic models rely on patients' gene information. In some cases (especially in clinics and hospitals who serve underrepresented populations), obtaining these information is impossible due to the lack of necessary equipment and lab tests. In such cases, clinical models and fixed-dose approaches are the only solutions for warfarin dosing. In other instances, even when it is possible to obtain the gene information from patients, the use of pharmacogenetic models might be questionable due to time constraints. For example, when a patient, whose gene information is not available, is involved in an accident and needs an immediate dose of warfarin, it might be unsafe to wait for the gene information to become available. It could take several hours before one can obtain the gene information by performing the required laboratory tests. For a patient involved in an accident this wait might result in death or serious blood clot complications.

## 5. Conclusions

The significance of prescribing an accurate initial dose for warfarin is undeniably important. Therefore several mathematical models have been proposed in order to predict the optimal dose for each patient. In this paper, a novel methodology for predicting the initial dose is proposed, which only relies on patients' clinical and demographic data. In this method, the patients are assigned to either one of two classes in the first phase. The patients who require doses of >30 mg/wk belong to the first class and the second class contains the patients who require doses of ≤30 mg/wk. This phase is implemented using relevance vector machines (RVM). Then, the optimal dose for each patient will be predicted using one of the two regression clinical models which are customized for each class. The proposed methodology outperformed two popular existing clinical prediction models (IWPC Cl and Gage Cl models), the method in [16], and the fixed-dose approach in terms of prediction accuracy. The methodology which is proposed in this work can be extended by investigating the best classifiers for patients of specific ethnicities.

## Figures and Tables

**Table 1:** Dataset description.

Continuous variables			

Target international normalized ratio	Mean		2.5
	Std. deviation		0.1
	Minimum		1.8
	Maximum		3.5

Body surface area	Mean		1.94
	Std. deviation		0.3
	Minimum		1.2
	Maximum		3.4

Categorical variables			

	Values	Frequency	Percent

Gender	0	1822	43.00%
	1	2415	57.00%

Race	1	2663	62.85%
	2	656	15.48%
	3	918	21.67%

Deep vein thrombosis and pulmonary embolism	0	3846	90.77%
	1	391	9.23%

Diabetes	0	3500	82.61%
	1	737	17.39%

Congestive heart failure	0	3492	82.42%
	1	745	17.58%

Valve replacement	0	3243	76.54%
	1	994	23.46%

Aspirin	0	3199	75.50%
	1	1038	24.50%

Simvastatin	0	3608	85.15%
	1	629	14.85%

Atorvastatin	0	3810	89.92%
	1	427	10.08%

Fluvastatin	0	4220	99.60%
	1	17	0.40%

Lovastatin	0	4153	98.02%
	1	84	1.98%

Pravastatin	0	4121	97.26%
	1	116	2.74%

Rosuvastatin	0	4208	99.32%
	1	29	0.68%

Amiodarone	0	3984	94.03%
	1	253	5.97%

Carbamazepine	0	4195	99.01%
	1	42	0.99%

Phenytoin	0	4197	99.06%
	1	40	0.94%

Rifampin	0	4231	99.86%
	1	6	0.14%

Sulfonamide Antibiotics	0	4214	99.46%
	1	23	0.54%

Macrolide antibiotics	0	4225	99.72%
	1	12	0.28%

Antifungal azoles	0	4210	99.36%
	1	27	0.64%

Smoker	0	3733	88.10%
	1	504	11.90%

Enzyme	0	4150	97.95%
	1	87	2.05%

Patient class	0	2111	49.82%
	1	2126	50.18%

Age	1	9	0.21%
	2	94	2.22%
	3	189	4.46%
	4	444	10.48%
	5	806	19.02%
	6	1023	24.14%
	7	1133	26.74%
	8	511	12.06%
	9	28	0.66%

**Table 2:** The confusion matrix.

Total accuracy		Actual values		
		Actual positive	Actual negative	
Predicted values	Predicted positive	True positives (TP)	False negatives (FN)	Precision+
	Predicted negative	False positives (FP)	True negative (TN)	Precision−
		Sensitivity	Specificity	

**Table 3:** Classification results for RVM.

Method	Accuracy	Sensitivity	Specificity	Precision+	Precision−
RVM	66%	63%	73%	81%	50%

**Table 4:** Comparing the prediction accuracy of the proposed methodology with IWPC Cl and Gage Cl models.

Methods	RMSE	MAE
The proposed methodology	11.6	8.4
IWPC Cl	13.8	9.1
Gage Cl	12.2	9.9
Sharabiani	18.1	12.7
Fixed-dose approach	18.7	12.3
